# Ultrasound-Attenuated Microorganisms Inoculated in Vegetable Beverages: Effect of Strains, Temperature, Ultrasound and Storage Conditions on the Performances of the Treatment

**DOI:** 10.3390/microorganisms8081219

**Published:** 2020-08-11

**Authors:** Daniela Campaniello, Maria Rosaria Corbo, Barbara Speranza, Milena Sinigaglia, Antonio Bevilacqua

**Affiliations:** Department of the Science of Agriculture, Food and Environment, University of Foggia, 71122 Foggia, Italy; daniela.campaniello@unifg.it (D.C.); mariarosaria.corbo@unifg.it (M.R.C.); barbara.speranza@unifg.it (B.S.); milena.sinigaglia@unifg.it (M.S.)

**Keywords:** beverage, probiotics, ultrasound, attenuation, carriers

## Abstract

Four microorganisms (*Lactobacillus acidophilus* LA5, *Bifidobacterium animalis* subsp. *lactis* DSM 10140 and *Lactiplantibacillus plantarum* c16 and c19) were attenuated through ultrasound (US) treatments (40% of power for 2, 4 and 6 min; and 60% for 2 min; pulses were set at 2 s) inoculated in rice–oats–almond–soy-based beverages and stored at 4 °C for eight days. All strains were able to survive throughout the storage independently by the food matrix. Concerning the effect on acidification, the results were analyzed through multifactorial analysis of variance (MANOVA) and the key-findings of this were: (i) The treatment with 40% of power for 6 min was the most efficient at delaying acidification; (ii) *Lb. acidophilus* LA5 showed the best capacity to delay acidification; (iii) in the soy-based beverage a lower acidification was found. In a second step, *L. plantarum* c16 and c19 were attenuated, inoculated in rice beverage, stored under a thermal abuse (for 4 and 24 h) and then at 4, 15 and 20 °C. The results showed that only when US were combined with refrigeration temperatures were they efficient at delaying acidification. Thus, a perspective for attenuation could be the optimization of the treatment to design an effective way to counteract acidification also under a thermal abuse.

## 1. Introduction

In the past, the function of foods was conceived only in relation to their ability to satisfy humans’ primary need to eat and provide the necessary nutrients. Nowadays, this concept has been extended to physical and mental well-being and functional foods satisfy this new concept. Functional foods are defined as “food or dietary components that may provide a health benefit beyond the basic function of providing nutrients” and in particular probiotic foods are the most promising sector [1].

It is known that probiotics (together with prebiotics and synbiotics, namely a combination of pre- and probiotics) are able to elicit changes in the gut biomass composition, improving and stimulating beneficial microflora [1,2].

Dairy products, and in detail, fresh milk and fermented drinks such as yogurt, are the most common probiotic vehicles; in fact, due to the fact that milk is rich in water, proteins and lipids, it acts as a protective matrix for probiotics from adverse stomach conditions. However, the growing health concerns related to lactose intolerance, allergy to milk proteins, the high cholesterol content as well as the high amounts of saturated fatty acids has directed consumers towards alternative foods to milk and its derivatives. In fact, in order to alleviate the drawbacks of dairy based probiotics, the development of non-dairy probiotic products, including food matrices from fruit, vegetables and cereals, has a promising future [3].

Fruit and vegetable beverages offer the possibility to incorporate nutrients and bioactive compounds, including vitamins, minerals, antioxidants, ω-3 and ω-6 fatty acids, fibers and plant extracts, prebiotics and probiotics. They represent the best-selling categories of functional foods, as they meet a wide range of consumer needs such as novelty, variety, naturalness, healthiness and packaging.

These beverages must be able to guarantee the survival of microorganisms during both processing and storage [1].

For example, carrot juice was a suitable medium for the growth of *Bifidobacterium lactis* and *Bifidobacterium bifidum*, and viable count remained at 10^8^ CFU/mL up to 24 h [4]. A strain of *Lactiplantibacillus plantarum* B28 was added in a beverage made from whole-grain oat and the viable cell counts at the end of the storage (21 days) were 10^6^–10^7^ CFU/mL.

Profir and Vizireanu [5] tested the viability of *Lactobacillus acidophilus*, *Lacticaseibacillus casei*, *Saccharomyces boulardii* in carrot, celery and beetroot juices and found that the lactic fermented juice showed a better stability during storage, with a good viability of probiotics.

Most of the microorganisms inoculated in foods (bifidobacteria and lactobacilli) are fermentative, and therefore producers of organic acids, which create hostile environments for pathogen proliferation. While this is a positive effect, because of their active metabolism, when probiotics or other microorganisms are added in food matrix, they could cause changes in the flavor and rheology of foods [6]. One way to overcome this problem could be attenuation through the use of ultrasound. The use of ultrasound (US) could delay acidification and counteract post-acidification in functional beverages improving their performances and some functional properties, without affecting the viability of strains [7,8]. A successful application of US (power of 50 to 80 W) in delaying acidification of *Limosilactobacillus reuteri, L. plantarum*, *Lc. casei*, bifidobacteria and propionibacteria both in organic rice beverage and laboratory media was obtained by Bevilacqua et al. [9] and Racioppo et al. [10].

On the other hand, viability could also be related to strain, physiological state, method used for culture preparation, oxygen level, presence of fibers and storage temperature [11].

Therefore, the aim of this study was to evaluate the suitability of US attenuation on four strains in order to counteract the acidification in some vegetable beverages, under both refrigeration and thermal abuse conditions, through two steps: (i) The evaluation of the effect of four different US treatments, for power and exposure time, on the survival of two *L. plantarum* (c16 and c19), *Lb. acidophilus* LA5 and *Bifidobacterium animalis* subsp. *lactis* 10140) strains inoculated in beverages based on almond, oats, rice and soy, stored for eight days at 4 °C, by also investigating the effect of US to delay beverage acidification; (ii) the effect of thermal abuse on the performance of attenuation.

## 2. Materials and Methods

### 2.1. Microorganisms

*Lactobacillus acidophilus* LA5 purchased from Chr. Hansen (Hørsholm, Denmark), *Bifidobacterium animalis* subsp. *lactis* DSM 10140 (Deutsche Sammlung von Mikroorganismen) and 2 strains of *Lactiplantibacillus plantarum* [12] named c16 and c19 [13] were used throughout this research.

Before each assay, the strains were cultured in an MRS broth (*L. plantarum* c16 and c19 at 30 °C for 48 h and *Lb. acidophilus* and *B. animalis* subsp. *lactis* at 37 °C for 24 and 48 h, respectively, under anaerobic conditions). Then, the cultures were centrifuged at 1200× *g* for 10 min; the supernatants were discarded and the pellet washed and suspended in sterile distilled water. The viable count of bacterial cultures was ca. 9 log CFU/mL for *L. plantarum* c16 and c19 and 7 log CFU/mL for *Lb. acidophilus* LA5 and *B. animalis subsp. lactis.*

### 2.2. Ultrasound Treatments

Bacterial suspensions were subjected to ultrasound (US) treatments through VC Vibra Cell Ultrasound equipment, model VC 130 (Sonics and Materials Inc., Newtown, CT, USA). The main variables of the treatment were the net power (40 and 60%) and the duration of the treatment (2, 4 and 6 min); pulse was set to 2 s. US combinations are listed in Table 1.

US equipment worked at a frequency–acoustic energy of 20 KHz–130 W, respectively, and the main efficiency of the probe was ca. 70%; thus, the net energy ranged from 36.4 to 54.6 W. Before each treatment, a washing of probe with ethanol and sterile distilled water was performed to counteract probable contaminations, while after sonication the samples were cooled on ice.

### 2.3. Viability and Acidification of Attenuated Strains in Vegetable Beverages

Four different commercial vegetable beverages were purchased from a local market, based on: Almonds, oats, rice and soy (pH, 6.30, 5.52, 6.64 and 7.1, respectively). Before the inoculation, beverages were analyzed to assess the absence of spoiling microorganisms through spread plating on Plate Count Agar (PCA, Oxoid, Basingstoke, UK) and Sabouraud Dextrose Agar (Oxoid).

Therefore, samples were inoculated to 7 log CFU/mL of *L. plantarum*, *B. animalis* subsp. *lactis* and *Lb. acidophilus* strains attenuated by US (see Section 2.2) and stored at 4 °C.

The viability of the microorganisms was assessed immediately after the treatment and throughout storage through the pour plate count on MRS Agar incubated at 30 °C for *L. plantarum* (c16 and c19) and at 37 °C for *Lb. acidophilus* LA5 and *B. animalis* subsp. *lactis* under anaerobic conditions. Untreated bacteria were used as controls. The analyses were performed over two different batches; for each batch the analyses were repeated twice.

During the storage time, the pH of each beverage was evaluated through a pH-meter Crison model micro pH 2001 (Crison, Barcellona, Spain) previously calibrated with two standard solutions at pH 4 and 7.02. Data from pH measurements were modeled as pH decreased. The experiments were performed in duplicate over two different batches.

The significant differences were pointed out by means of multifactorial analysis of variance (MANOVA) and Tukey’s test as the post hoc comparison test (*p* < 0.05); the strain, the combinations, the kind of beverage and the time were used as categorical predictors, while acidification (that is pH decrease) was used as the dependent variable. Statistics were done through the software Statistica for Windows ver 12.0 (Statsoft, Tulsa, OK, USA).

### 2.4. Effect of Thermal Abuse on Lactiplantibacillus plantarum c16 and c19 Attenuated by US and Inoculated in Rice Beverage

Samples of rice beverages were separately inoculated to 7 log CFU/mL of *L. plantarum* c16 and c19 attenuated by US treatment (40% of power for 6 min and impulses of 2 s), incubated at 30 °C (thermal abuse) for 4 and 24 h and then stored at 4, 15 and 20 °C. Table 2 reports the thermal and US treatments applied and the code used for each sample.

**Figure 1:**
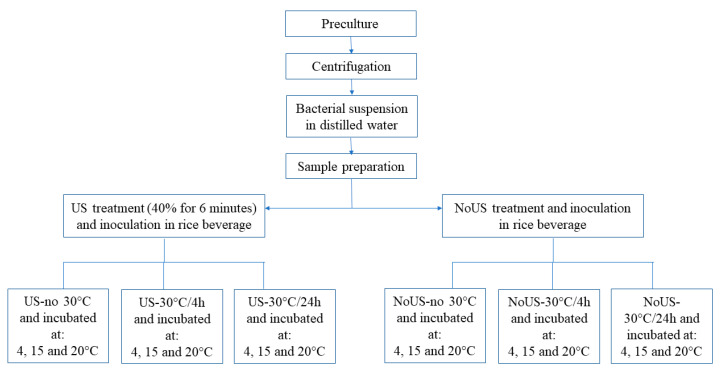
Flow chart for sample preparation under thermal abuse.

Aliquots of beverage inoculated with untreated microorganisms were used as controls. Sample preparation is shown in Figure 1.

Cell viability and pH were assessed throughout time; the analyses were performed over two different batches. The results were analyzed by means of MANOVA. Strains, temperatures of storage, treatments (US and thermal abuse) and time were used as categorical predictors, while acidification (that is pH decrease) was used as the dependent variable.

## 3. Results

### 3.1. Viability and Acidifying Capability of Attenuated Strains in Vegetable Beverages

The first experiment was aimed at assessing the viability of microorganisms after US treatments and their effect on beverage acidification. *Lb. acidophilus* LA5, *L. plantarum* c16 and c19, and *B. animalis* subsp. *lactis* were subjected to four different US treatments (see Table 1), inoculated in four different vegetable beverages (based on rice, oats, almond and soy) and stored at 4 °C for eight days.

The viability was not significantly affected by US (*p* > 0.05); in fact, immediately after the treatments the viable count of all strains was ≥ 7 log CFU/mL and it did not experience a significant decrease throughout the time (*p* > 0.05) (see Table S1).

Regarding the effect on acidification, the results were analyzed through the MANOVA approach to evaluate the effect of strains, combinations, beverage and time on acidification, expressed as pH decrease (DpH), and the outputs are the standardized effects and the decomposition of the statistical hypothesis.

Table 3 shows the standardized effects. Strains, combinations, beverages and time were significant both as individual and interactive terms; however, their statistical weights were different. The most important factor, as a single term, was time (*F* = 4835.34), followed by beverage (*F* = 1493.53), combinations and strains (*F* = 170.54 and 151.75, respectively). The most important interactive term was “strains x beverages” (*F* = 383.94).

The MANOVA approach, through the table of the standardized effects, offers a qualitative estimation of each factor (strains, combinations, beverages and time) without focusing on how much each predictor affects the dependent variable (acidification).

**Figure 2:**
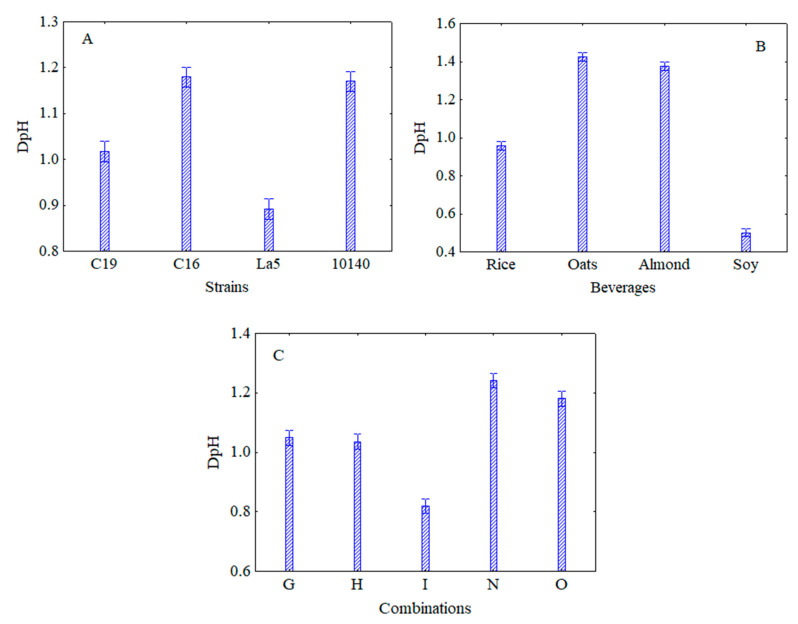
Decomposition of the statistical hypothesis for the individual terms of strains (**A**), beverages (**B**) and combinations (**C**) on acidification. Bars denote 95% confidence intervals. For the code of US treatments see Table 1.

This information can be found in the following figures. In Figure 2 the decomposition of the statistical hypothesis for the individual terms of strains (A), beverages (B) and combinations (C) is shown. The lowest acidification was found for *Lb. acidophilus* LA5 (DpH ca. 0.90), while the highest pH decrease was recovered in the samples containing either *B. animalis* subsp. *lactis* 10140 and *L. plantarum* c16 (DpH, 1.20) (Figure 2A).

The effects of beverages are shown in Figure 2B. The lowest DpH was found in the soy-based beverage (DpH = 0.5), although good results were also observed for the rice-based one (DpH ca. 0.95).

Figure 2C shows the effect of US treatment; the lowest DpH was found for the combination I (40% of power for 6 min) (DpH, 0.82), while the highest one in the combination O (60% for 2 min) (DpH, 1.2); G and H (40% of power for 2 and 4 min, respectively) showed an intermediated trend.

**Figure 3:**
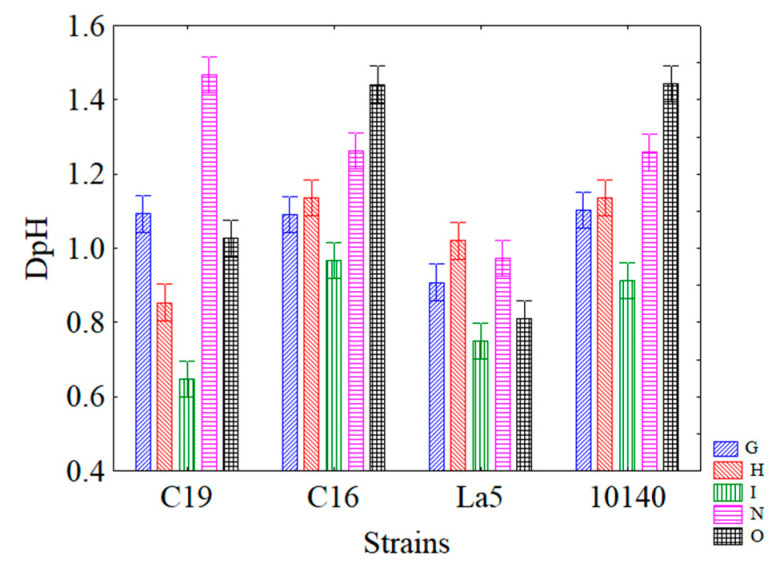
Decomposition of the statistical hypothesis for the interaction strains x combinations on acidification. Bars denote 95% confidence intervals.

Figure 3 reports the interaction strain x combination and shows that combination I was the best for each of the tested strains: The lowest acidification was observed for *L. plantarum* c19 (DpH ca. 0.65), followed by *Lb. acidophilus* LA5 (DpH ca. 0.75), *B. animalis* subsp. *lactis* (DpH ca. 0.9) and *L. plantarum* c16 (DpH ca. 0.98).

**Figure 4:**
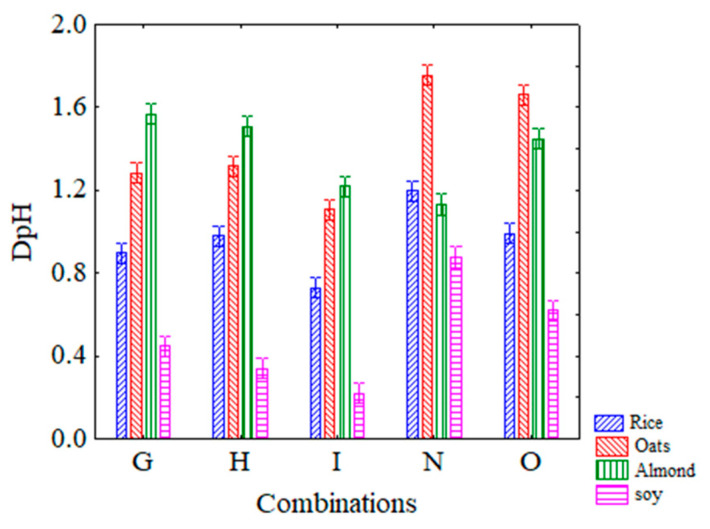
Decomposition of the statistical hypothesis for the interaction combinations vs beverages on acidification. Bars denote 95% confidence intervals. For the code of US treatments see Table 1.

Through the interaction combination vs beverages (Figure 4), soy and almond-based beverages resulted in the best and the worst performers with the DpH values ranging from 0.2–0.6 and 1.2–1.7, respectively, for all US treatments (combination G, H, I and O).

**Figure 5:**
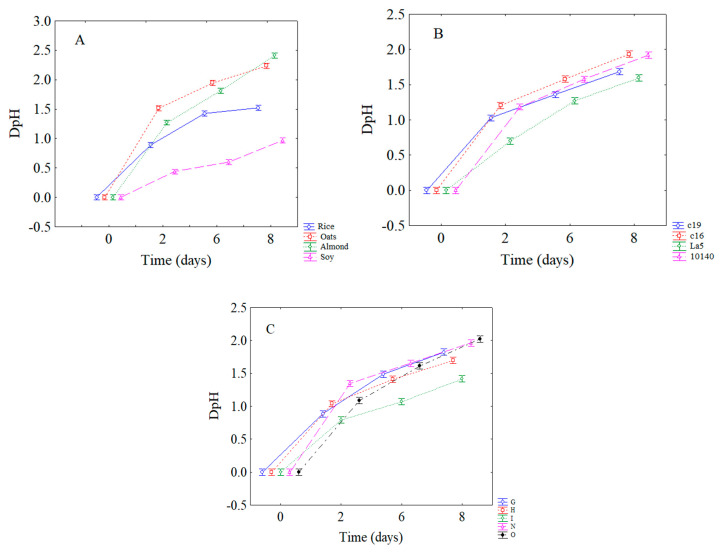
Decomposition of the statistical hypothesis for the interaction of time vs beverages (**A**), vs strains (**B**) and vs combinations (**C**) on acidification. Bars denote 95% confidence intervals. For the code of US treatments see Table 1.

As reported in Table 3, time exerted the strongest effect on acidification; thus, it was analyzed in interaction with the other variables; Figure 5 shows the decomposition of the statistical hypothesis for the interaction of time vs beverages (Figure 5A), vs strains (Figure 5B) and vs combinations (Figure 5C). Acidification increased throughout time, but the soy-based beverage (Figure 5A), *Lb. acidophilus* LA5 (Figure 5B), and combination I (Figure 5C) showed the minor DpH values.

### 3.2. Effect of Thermal Abuse on Lactiplantibacillus plantarum c16 and c19 Attenuated by US and Inoculated in Rice Beverage

The second step of this research aimed at assessing the effect of thermal abuse (30 °C for 4 and 24 h) on attenuated *L. plantarum* c16 and c19, inoculated in rice beverage and stored at 4, 15 and 20 °C. The US treatment applied was 40% power for 6 min and impulse at 2 s (corresponding to combination I). Untreated sample were used as controls.

The effects of strain, temperature, treatment (US and thermal abuse) and time on acidification are shown in Table 4. Time was the most significant term followed by temperature, treatment and strain (*F* = 7673.6, 2159.2, 520.5 and 66.2, respectively), while the most important interactive term was time*strain (*F* = 190.3).

**Figure 6:**
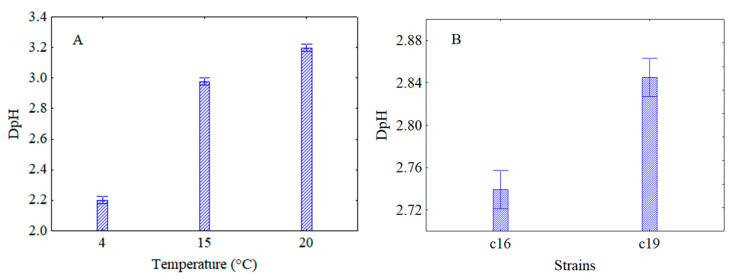
Decomposition of the statistical hypothesis for the effects of temperature of storage (**A**) and strains (**B**) on acidification. Bars denote 95% confidence intervals. Experiment on the effect of a thermal abuse in rice beverage for *L. plantarum* c16 and c19.

The decomposition of the statistical hypothesis pointed out the effects of storage temperature (Figure 6A) and strains (Figure 6B) on acidification. Figure 6A shows that refrigeration exerted the best controlling effect on acidification; in fact, at 4 °C the DpH value (2.2) was at least one unit lower than at 20 °C (3.2), while, as Figure 6B shows, the acidification was similar for both strains (2.74–2.86).

**Figure 7:**
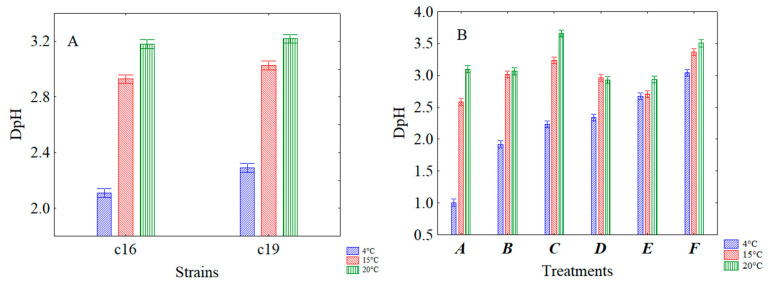
Decomposition of the statistical hypothesis for the interaction of the temperature vs strains (**A**) and vs treatments (**B**) on acidification. Bars denote 95% confidence intervals. Experiment on the effect of a thermal abuse in rice beverage for *L. plantarum* c16 and c19. For the code of US treatments see Table 2.

Through the interaction of the temperature vs strain (Figure 7A) and vs treatment (Figure 7B) it was found that the storage at 4 °C exerted a better effect on acidification for both strains (Figure 7A) and for each of the treatments carried out (Figure 7B). In fact, at 4 °C, DpH values were from 2.1 to 2.3 for *L. plantarum* c16 and c19, respectively. At 15 and 20 °C, higher DpH values were observed (of ca. 3 and 3.2, respectively) (Figure 7A). Concerning the interaction temperature vs treatments, lower acidification (DpH = 1) was obtained when the microorganisms were attenuated but not subjected to the thermal abuse (treatment A) and stored at 4 °C.

**Figure 8:**
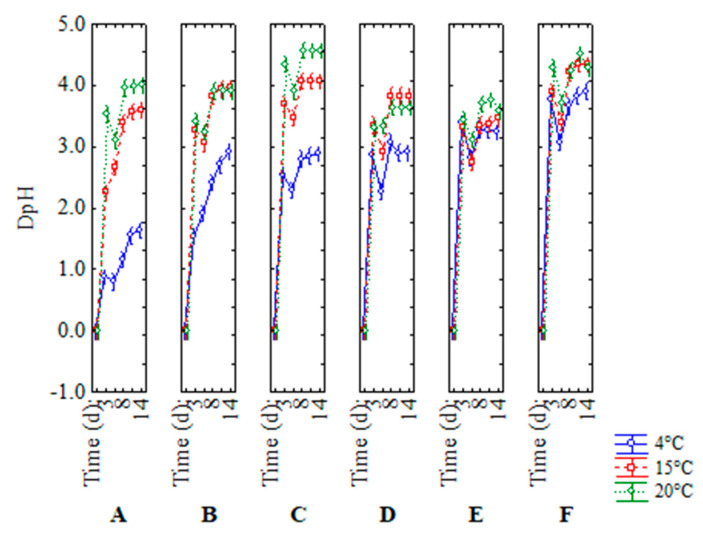
Decomposition of the statistical hypothesis for the interaction temperature–time–treatment on acidification. Bars denote 95% confidence intervals. Experiment on the effect of a thermal abuse in rice beverage for *L. plantarum* c16 and c19. For the code of US treatments see Table 2.

Finally, Figure 8 (temperature–time–treatment interaction) points out that the best condition to delay acidification was in correspondence to treatment A at 4 °C for both strains c16 and c19. In fact, DpH values were clearly lower than all other treatments; for example, compared to its control (B treatment), which had a DpH ranging from 0 to 3, treatment A showed a DpH of 0–1.5, while acidification occurred in experiments C and E (attenuated strains in beverages stored under a thermal abuse) without differences to their controls (samples D and F).

## 4. Discussion

Vegetable beverages are considered an ideal alternative to dairy products and they have proven to be good carriers of probiotics and functional microorganisms, although the maintenance of their viability is more difficult in non-dairy than in dairy matrices [14].

However, microorganisms generally retain an active metabolism in some products and when they are inoculated in high-pH matrices they tend to metabolize sugars and to reduce pH, thus leading to unfavorable changes in sensory scores; this phenomenon is called over- or post-acidification [8]. A technological solution to this challenge is attenuation, that is a modulation of the technological performances of probiotics and other microorganisms [7,8,9]. Several approaches can be used; among others US and high-pressure homogenization showed promising performance in food [7]. Namely, US could exert different effects (inactivation, attenuation and modulation of metabolism, metabolism enhancement), depending on several processing parameters, like power, pulse and duration of the treatment [15,16].

Generally, at the time of consumption, 10^6^–10^7^ colony-forming units (CFU) per mL or g of food are required to favor probiotic survival throughout gastrointestinal digestion; therefore, the maintenance of high concentrations during processing and storage is important [14]. US treatment fulfilled this requisite because the viability was never affected; this result was also found in a previous research on two strains (*Lc. casei* LC01 and *B. animalis* subsp. *lactis* Bb-12) inoculated in an organic rice beverage [9]. However, strain specificity and power/pulse could play a significant role and cause a detrimental effect on viability. Yeo and Liong [17] focused on the effect of US on the growth of bifidobacteria (*Bifidobacterium* FTDC 8943, *B. longum* FTDC8643) and lactobacilli (*Lactobacillus* sp. FTDC2113, and *Lc. casei* ATCC393) inoculated in soymilk, and they reported that, immediately after the treatment, the growth of target strains was significantly decreased (*p* < 0.05), probably due to membrane permeabilization, cell lysis and membrane lipid peroxidation. Then, the growth kinetic of cells was similar to the control because cells repaired injury.

The same phenomena probably occurred also in our samples. According to Ojha et al. [18] US acts on cells through a complex mechanism called sonoporation, responsible for the release of components from cells, mechanic shock and transient or permanent injury. Attenuation could be the result of a sub-lethal injury, as well as of a transient impairment between viability and metabolism. The idea of transient impairment was confirmed by the evidence that attenuated strains acquired an active metabolism throughout storage.

All tested treatments were able to delay acidification, exerting a positive effect on the reduction of acidification of strains, but the best performances were observed in correspondence to 40% power for 6 min (combination I) probably due to the significant role of the duration of the treatment. Among the vegetable beverages tested, the soy-based beverage experienced the lowest acidification rate, although good results were also observed for the rice-based beverages. This result can be explained in the composition of soy-based beverages that, unlike cereal-based beverages, are rich in proteins but low in carbohydrates [19].

In some papers published in the past on the same topic, the best combination for attenuation was different from that recovered in this research. Racioppo et al. [10] applied US (power ranging from 40 to 80%; time ranging from 2 to 6 min; pulses set to 2 s) for the attenuation of *Li. reuteri, L. plantarum, B. longum and B. infantis* and they found that the best combination to avoid acidification was the treatment at 60% power for 6 min. Then, Bevilacqua et al. [20] attenuated *Acidipropionibacterium jensenii* and *Propionibacterium freudenreichii* by using US at different power levels (40 and 60%) and treatment times (4, 6 and 8 min) and they found that the best combinations were 40% power for 8 min (*A. jensenii*) and 60% power for 4 min (*P. freudenreichii* subsp. *freudenreichii*). The different levels of power and time were probably the result of a strain dependence of treatment, as also suggested by the difference between *L. plantarum* c16 and *L. plantarum* c19 in this research, as well as by the different suspension mediums (water, broth or complex media).

The second part of this research focused on the maintenance of attenuation if a thermal abuse occurred. In fact, the rigorous maintenance of the cold chain would be desirable for food safety or to ensure probiotic survival; however, it is not always respected. Therefore, the rice-based beverage was inoculated with the two strains of *L. plantarum* attenuated through the treatment at 40% power for 6 min (combination I) and incubated under thermal abuse at 30 °C for 4 (short abuse) and 24 h (long-term abuse). The results suggested that attenuation should be combined with refrigeration at least for the combinations used in this research, probably as a result of the technological robustness of *L. plantarum*, which at high temperatures would be able to repair injuries and acquire an active metabolism. As reported above, in fact, US could cause a transient permeabilization of membrane; however, this effect was reversible and the microorganisms tend to restore their homeostasis. This ability was enhanced under thermal abuse (higher storage temperature) because this ability to restore cells is probably fast at high temperatures.

On the other hand, attenuation was able to control acidification by *Lc. casei* LC01 in a rice beverage also after a thermal abuse of 4 h [9], thus confirming the idea of a strong strain dependence of the treatment.

Therefore, further investigations are necessary to evaluate if by also improving the method (for example, using a multi-step treatment) for *L. plantarum* it would be possible to control acidification in conditions of thermal abuse.

## 5. Conclusions

The results of this research confirm that a US treatment could be successfully applied to counteract the regrettable problem of acidification, allowing the creation of new formulations for food industries. Considering the viability maintenance for eight days at 4 °C, all vegetable beverages (oat, soy, rice, almond) were good carriers for attenuated microorganisms, although the best results, in terms of reduction of acidification, were observed in the soy-based beverage. In addition, among the strains, *Lb. acidophilus* LA5 showed the best outcome. This research also simulated a hypothetical situation in which the cold chain was not respected (thermal abuse); however, it was found that in our conditions strict refrigeration should be maintained for a duration of the effects of attenuation. Thus, a perspective for attenuation could be the optimization of the treatment to design an effective way to counteract acidification also under thermal abuse.

## Figures and Tables

**Table 1:** Ultrasound (US) treatments.

Combinations	Power (%)	Time (Minutes)	Pulse (Seconds)
**G**	40	2	2
**H**	40	4	2
**I**	40	6	2
**O**	60	2	2
**N (Control)**	-	-	-

**Table 2:** Sample codes; experiment on thermal abuse.

Code	Treatments
*A*	US treatment
*B*	Control
*C*	US treatment; storage at 30 °C for 4 h
*D*	Control; storage at 30 °C for 4 h
*E*	US treatment; storage at 30 °C for 24 h
*F*	Control; storage at 30 °C for 24 h

**Table 3:** Standardized effects of beverages, combinations and time on acidification. The results were obtained by multifactorial analysis of variance (MANOVA) and Tukey’s test (*p* < 0.05) as the post hoc comparison test.

	*SS*	Degree of Freedom	*MS*	*F*	*p*
Intercept	725.69	1	725.69	36,596.77	0.00
{1} strains	9.027	3	3.01	151.75	0.00
{2} combinations	13.53	4	3.38	170.54	0.00
{3} beverages	88.85	3	29.62	1493.53	0.00
{4} time	287.64	3	95.88	4835.34	0.00
Strains x combinations	9.12	12	0.76	38.31	0.00
Strains x beverages	68.52	9	7.61	383.94	0.00
Combinations x beverages	12.69	12	1.06	53.32	0.00
Strains x time	3.86	9	0.43	21.65	0.00
Combinations x time	6.66	12	0.56	28.01	0.00
Beverages x time	35.55	9	3.95	199.19	0.00
Strains x combinations x beverages	19.99	36	0.55	28.01	0.00
Strains x combinations x time	4.87	36	0.13	6.82	0.00
Strains x beverages x time	35.26	27	1.31	65.86	0.00
Combinations x beverages x time	10.22	36	0.28	14.32	0.00
1 × 2 × 3 × 4	21.66	108	0.20	10.11	0.00
Error	6.34	320	0.02		

*SS*, sum of squares; *MS*, mean square residual; *F*, Fisher test.

**Table 4:** Standardized effects of strains, storage temperature, treatments (US and thermal abuse, see Table 2) and time on acidification. The results were obtained by MANOVA (analyses of variance) and Tukey’s test (*p* < 0.05) as the post hoc comparison test.

	*SS*	Degr. of Freedom	*MS*	*F*	*p*
Intercept	3368.37	1	3368.37	184,038.30	0.00
{1}temperature	79.04	2	39.52	2159.20	0.00
{2}time	702.23	5	140.45	7673.60	0.00
{3}treatment	47.63	5	9.53	520.50	0.00
{4}strains	1.21	1	1.21	66.20	0.00
Temperature x time	16.79	10	1.68	91.70	0.00
Temperature x treatment	33.49	10	3.35	183.00	0.00
Time x treatment	14.28	25	0.57	31.20	0.00
Temperature x strains	0.36	2	0.18	9.90	0.00
Time x strains	17.41	5	3.48	190.30	0.00
Treatment x strains	11.97	5	2.39	130.80	0.00
Temperature x time x treatment	9.97	50	0.20	10.90	0.00
Temperature x time x strains	1.14	10	0.11	6.20	0.00
Temperature x treatment x strains	1.58	10	0.16	8.60	0.00
Time x treatment x strains	4.96	25	0.20	10.80	0.00
1 × 2 × 3 × 4	3.20	50	0.06	3.50	0.00
Error	3.95	216	0.02		

*SS*, sum of squares; *MS*, mean square residual; *F*, Fisher test.

## Supplementary Materials

The following are available online at https://www.mdpi.com/2076-2607/8/8/1219/s1, Table S1: microbial cell load (log CFU/ mL) of *Lactiplantibacillus plantarum* c19 and c16, *Lactobacillus acidophilus* La5 and *Bifidobacterium animalis* subsp. *lactis* DSM 10140 attenuated through the combinations G, H, I and O (N = control), inoculated in vegetable beverages and stored at 4 °C for 8 days. Data were expressed as mean ± standard deviation.

## Abbreviations

*Lb.*	*Lactobacillus*
*L.*	*Lactiplantibacillus*
*Lc.*	*Lacticaseibacillus*
*Li.*	*Limosilactobacillus*
