# Supplementary material for: Ultrasound-Attenuated Microorganisms Inoculated in Vegetable Beverages: Effect of Strains, Temperature, Ultrasound and Storage Conditions on the Performances of the Treatment
Source: Microorganisms. 2020 Aug 11;8(8):1219. doi: 10.3390/microorganisms8081219 (PMC7464006; doi:10.3390/microorganisms8081219)
Supplement: Supplementary file 1 [file microorganisms-08-01219-s001.pdf]

Table S1: microbial cell load (log CFU/ mL) of *Lactiplantibacillus plantarum* c19 and c16, *Lactobacillus acidophilus* La5 and *Bifidobacterium animalis* subsp. *lactis* DSM 10140 attenuated through the combinations G, H, I and O (N = control), inoculated in vegetable beverages and stored at 4°C for 8 days. Data were expressed as mean ± standard deviation.

	***Lactiplantibacillus plantarum* C19**					
Beverages	***Time (days)***	***Combinations***				
		G	***H***	***I***	***N***	***O***
**RICE**	*0*	7.47 ± 0.00	7.13 ± 0.00	7.00 ± 0.03	7.11 ± 0.22	7.89 ± 0.00
	2	6.79 ± 0.87	7.03 ± 0.53	7.00 ± 0.00	7.07 ± 0.17	7.06 ± 0.00
	6	7.26 ± 0.37	6.97 ± 0.78	7.10 ± 0.05	7.38 ± 0.29	7.64 ± 0.11
	8	6.74 ± 0.07	6.97 ± 0.81	6.95 ± 0.06	7.44 ± 0.68	7.39 ± 0.12
**OATS**	*0*	7.47 ± 0.00	7.13 ± 0.00	7.00 ± 0.03	7.13 ± 0.05	7.89 ± 0.00
	2	6.83 ± 0.32	7.11 ± 0.05	6.90 ± 0.07	6.95 ± 0.46	6.86 ± 0.20
	6	7.35 ± 0.26	7.38 ± 0.25	7.12 ± 0.00	7.73 ± 0.16	7.21 ± 0.15
	8	7.25 ± 0.07	7.33 ± 0.04	7.25 ± 0.11	7.26 ± 0.03	7.31 ± 0.21
**SOY**	*0*	7.47 ± 0.00	7.13 ± 0.00	7.00 ± 0.03	7.12 ± 0.21	7.89 ± 0.00
	2	6.81 ± 0.02	7.05 ± 0.02	6.75 ± 0.13	7.02 ± 0.02	6.80 ± 0.11
	6	7.90 ± 0.03	7.28 ± 0.63	7.03 ± 0.03	7.99 ± 0.11	7.43 ± 0.05
	8	7.60 ± 0.26	7.41 ± 0.57	7.11 ± 0.13	7.91 ± 0.01	7.48 ± 0.25
**ALMOND**	*0*	7.47 ± 0.00	7.13 ± 0.00	7.00 ± 0.03	7.23± 0.00	7.89 ± 0.00
	2	7.79 ± 0.27	7.20 ± 0.12	7.16 ± 0.12	7.46 ± 0.45	7.65 ± 0.19
	6	7.74 ± 0.14	7.18 ± 0.33	7.20 ± 0.00	7.65 ± 0.10	7.57 ± 0.08
	8	7.74 ± 0.06	7.00 ± 0.00	6.85 ± 0.16	6.88 ± 0.59	7.24 ± 0.76

	Lactiplantibacillus plantarum C16					
Beverages	***Time (days)***	***Combinations***				
		G	***H***	***I***	***N***	***O***
**RICE**	*0*	7.48 ± 0.00	7.40 ± 0.00	7.20 ± 0.00	7.23 ± 0.09	7.88 ± 0.00
	2	6.79 ± 0.86	7.07 ± 0.46	6.90 ± 0.07	7.07 ± 0.16	7.06 ± 0.00
	6	7.26 ± 0.37	6.97 ± 0.78	7.00 ± 0.00	7.27 ± 0.38	7.62 ± 0.11
	8	6.76 ± 0.08	6.89 ± 0.83	7.15 ± 0.10	7.35 ± 0.49	7.39 ± 0.12
**OATS**	*0*	7.48 ± 0.00	7.40 ± 0.00	7.20 ± 0.00	7.08 ± 0.10	7.88 ± 0.00
	2	6.85 ± 0.21	7.09 ± 0.12	6.92 ± 0.13	6.98 ± 0.48	6.91 ±0.13
	6	7.27 ± 0.38	7.36 ± 0.26	7.11 ± 0.08	7.69 ± 0.07	7.24 ± 0.09
	8	7.25 ± 0.08	7.35 ± 0.07	7.10 ± 0.02	7.22 ± 0.04	7.26 ± 0.20
**SOY**	*0*	7.48 ± 0.00	7.40 ± 0.00	7.20 ± 0.00	7.13 ± 0.10	7.88 ± 0.00
	2	6.96 ± 0.06	7.19 ± 0.28	6.95 ± 0.12	7.23 ± 0.07	7.32 ± 0.59
	6	7.68 ± 0.29	7.36 ± 0.64	7.13 ± 0.14	7.90 ± 0.25	7.40 ± 0.00
	8	7.59 ± 0.17	7.37 ± 0.52	7.22 ± 0.08	7.85 ± 0.09	7.50 ± 0.14
**ALMOND**	*0*	7.48 ± 0.00	7.40 ± 0.00	7.20 ± 0.00	7.07 ± 0.11	7.88 ± 0.00
	2	7.60 ± 0.00	7.15 ± 0.21	7.00 ± 0.00	7.35 ± 0.50	7.61 ± 0.19
	6	7.67 ± 0.18	7.10 ± 0.11	6.93 ± 0.11	7.65 ± 0.07	7.56 ± 0.11
	8	7.70 ± 0.00	7.00 ± 0.00	6.85 ± 0.25	6.89 ± 0.58	7.27 ± 0.60

Beverages	Time (days)	*Lactobacillus acidophilus* La5				
		Combinations				
		G	*H*	*I*	*N*	*O*
**RICE**	*0*	7.04 ± 0.00	7.51 ± 0.00	7.20 ± 0.04	7.36 ± 0.00	7.57 ± 0.00
	2	7.45 ± 0.08	7.60 ± 0.05	7.22 ± 0.06	7.49 ± 0.00	7.29 ± 0.07
	6	7.27 ± 0.04	7.63 ± 0.04	7.25 ± 0.01	7.79 ± 0.06	7.36 ± 0.17
	8	7.07 ± 0.09	7.56 ± 0.04	7.21 ± 0.11	7.73 ± 0.03	7.28 ± 0.15
**OATS**	*0*	7.04 ± 0.00	7.51 ± 0.00	7.20 ± 0.04	7.36 ± 0.00	7.57 ± 0.00
	2	7.58 ± 0.03	7.60 ± 0.28	7.30 ± 0.02	7.44 ± 0.02	7.60 ± 0.29
	6	7.56 ± 0.02	7.55 ± 0.23	7.18 ± 0.09	7.35 ± 0.07	7.52 ± 0.11
	8	7.49 ± 0.01	7.55 ± 0.21	7.10 ± 0.03	7.30 ± 0.00	7.55 ± 0.11
**SOY**	*0*	7.04 ± 0.00	7.51 ± 0.00	7.20 ± 0.04	7.36 ± 0.00	7.57 ± 0.00
	2	7.54 ± 0.08	7.30 ± 0.15	7.25 ± 0.10	7.54 ± 0.16	7.73 ± 0.32
	6	7.13 ± 0.10	7.10 ± 0.00	7.21 ± 0.07	6.90 ± 0.17	7.32 ± 0.45
	8	7.52 ± 0.26	7.25 ± 0.11	7.00 ± 0.18	7.25 ± 0.32	7.27 ± 0.45
**ALMOND**	*0*	7.04 ± 0.00	7.51 ± 0.00	7.20 ± 0.04	7.36 ± 0.00	7.57 ± 0.00
	2	7.40 ± 0.06	7.51 ± 0.05	7.30 ± 0.09	7.70 ± 0.07	7.42 ± 0.15
	6	7.09 ± 0.19	7.52 ± 0.26	7.10 ± 0.02	7.18 ± 0.12	6.97 ± 0.38
	8	7.26 ± 0.12	7.45 ± 0.07	7.00 ± 0.00	7.41 ± 0.01	7.15 ± 0.03

Beverages	Time (days)	*Bifidobacterium animalis* subsp. *lactis* DSM 10140				
		Combinations				
		G	*H*	*I*	*N*	*O*
**RICE**	*0*	7.43 ± 0.00	7.06 ± 0.00	7.00 ± 0.00	7.29 ± 0.00	7.22 ± 0.00
	2	7.17 ± 0.00	7.29 ± 0.00	7.10 ± 0.01	7.26 ± 0.00	7.23 ± 0.00
	6	7.33 ± 0.00	7.47 ± 0.00	7.22 ± 0.04	7.69 ± 0.10	7.59 ± 0.00
	8	7.32 ± 0.00	7.40 ± 0.00	7.11 ± 0.05	7.50 ± 0.10	7.48 ± 0.00
**OATS**	*0*	7.43 ± 0.00	7.06 ± 0.00	7.00 ± 0.00	7.29 ± 0.00	7.22 ± 0.00
	2	7.44 ± 0.10	7.47 ± 0.30	7.13 ± 0.10	7.52 ± 0.40	7.49 ± 0.40
	6	6.72 ± 0.10	6.54 ± 0.20	6.40 ± 0.21	7.33 ± 0.50	7.70 ± 0.10
	8	6.65 ± 0.10	6.52 ± 0.10	6.41 ± 0.10	7.42 ± 0.20	7.70 ± 0.10
**SOY**	*0*	7.43 ± 0.00	7.06 ± 0.00	7.00 ± 0.00	7.29 ± 0.00	7.22 ± 0.00
	2	7.15 ± 0.00	7.13 ± 0.10	7.00 ± 0.00	7.54 ± 0.40	7.42 ± 0.30
	6	7.33 ± 0.10	7.45 ± 0.00	7.18 ± 0.12	7.40 ± 0.50	7.73 ± 0.40
	8	7.30 ± 0.00	7.42 ± 0.00	7.20 ± 0.13	7.54 ± 0.10	7.69 ± 0.00
**ALMOND**	*0*	7.43 ± 0.00	7.06 ± 0.00	7.00 ± 0.00	7.29 ± 0.00	7.22 ± 0.00
	2	7.23 ± 0.00	7.34 ± 0.10	7.10 ± 0.09	7.28 ± 0.00	7.20 ± 0.10
	6	7.21 ± 0.40	7.50 ± 0.00	7.18 ± 0.05	7.32 ± 0.00	7.21 ± 0.20
	8	7.20 ± 0.30	7.39 ± 0.10	7.00 ± 0.08	7.38 ± 0.10	7.20 ± 0.10
